# Supplementary figures and images for: Development of a novel Francisella tularensis Live Vaccine Strain expressing ovalbumin provides insight into antigen-specific CD8+ T cell responses
Source: PLoS One. 2017 Dec 28;12(12):e0190384. doi: 10.1371/journal.pone.0190384 (PMC5746256; doi:10.1371/journal.pone.0190384)

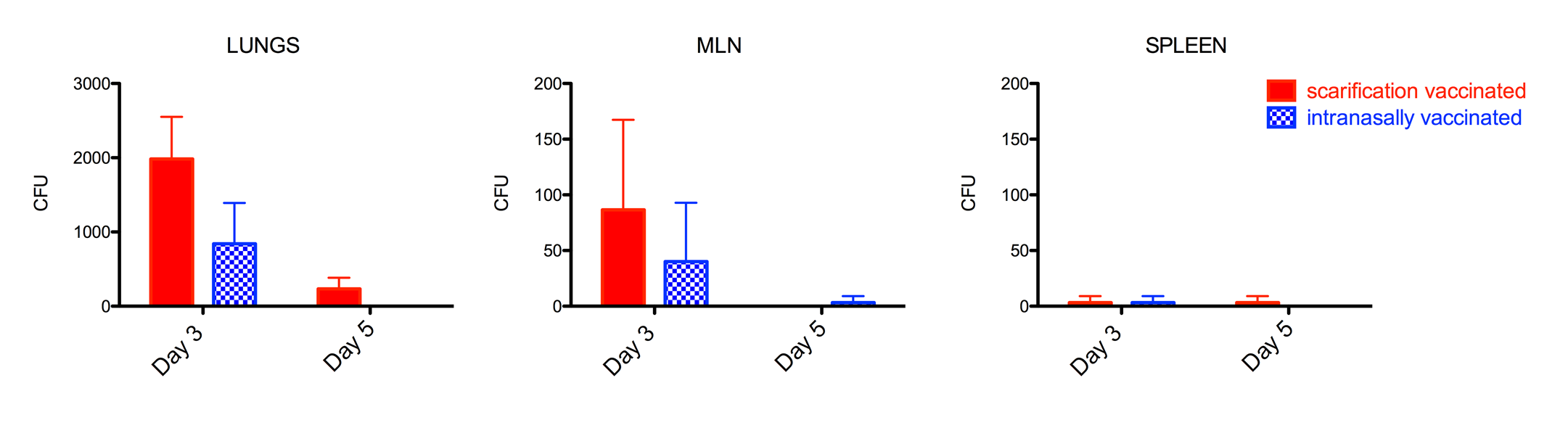

Supplement: S1 Fig — Mice (n = 3 per group) were given a priming vaccination of LVS-OVA via the scarification or intranasal route, followed by a booster with 10,000 CFU on day 30. Bacterial burdens are shown on day 3 and day 5 after the booster. (TIF) [file pone.0190384.s001.tif]
